# Primary hepatic mucosa-associated lymphoid tissue lymphoma complicated with atrial fibrillation: A case report and literature review

**DOI:** 10.1097/MD.0000000000036926

**Published:** 2024-01-12

**Authors:** Chenming Liu, Yuxing Liu, Jiayao Zhang, Yingjie Chai, Baochun Lu, Haijun Tang

**Affiliations:** a Department of Hepatobiliary and Pancreatic Surgery, Shaoxing People’s Hospital, Shaoxing, China; b Zhejiang University School of Medicine, Hangzhou, China; c Department of Colorectal and Anal Surgery, Jinhua Hospital, Zhejiang University School of Medicine, Jinhua, China; d Department of Hepatobiliary Surgery, Haining People’s Hospital, Jiaxing, China.

**Keywords:** atrial fibrillation, primary hepatic mucosa-associated lymphoid tissue lymphoma, radiological image

## Abstract

**Rationale:**

Primary hepatic mucosa-associated lymphoid tissue (MALT) lymphoma is a rare malignant primary hepatic lymphoma. The sensible choice of treatment for patients with primary lymphoma combined with atrial fibrillation (AF) is controversial and challenging.

**Patient concerns:**

The patient presented with both primary hepatic MALT lymphoma and AF, which was difficult to manage.

**Diagnoses:**

Pathological and immunohistochemical examination are helpful for definitive diagnosis.

**Interventions:**

Surgical resection and subsequent anticoagulant therapy are main treatment methods, and adjuvant therapy depends on the situation.

**Outcomes:**

Primary hepatic MALT lymphoma is easy to misdiagnosis due to a lack of typical symptoms and imaging signs.

**Lessons:**

This case highlights for patients with primary hepatic MALT lymphoma combined with AF, toxicity caused by adjuvant chemotherapy should be fully considered, and careful selection should be made based on the general conditions and complications of patients.

## 1. Introduction

Primary hepatic lymphoma (PHL) is a rare extranodal malignant lymphoma originating from liver lymphatic tissue, accounting for only 1% of all malignant lymphomas.^[1]^ Mucosa-associated lymphoid tissue (MALT) is a low-grade malignant and inert B-cell non-Hodgkin lymphoma, first described by Isaacson and Wright in 1983, which has an even lower incidence.^[2]^ Primary hepatic MALT is a subtype of PHL and its etiology is unclear. Atrial fibrillation (AF) is the most serious disorder of atrial electrical activity and one of the common tachyarrhythmias. According to the latest literature, there has been little research linking the 2 seemingly unrelevant diseases. Here we present a case of primary hepatic MALT lymphoma complicated with AF. At the same time, we review relevant published literature to discuss the diagnosis and treatment of this rare condition.

## 2. Case report

**Figure 1.**
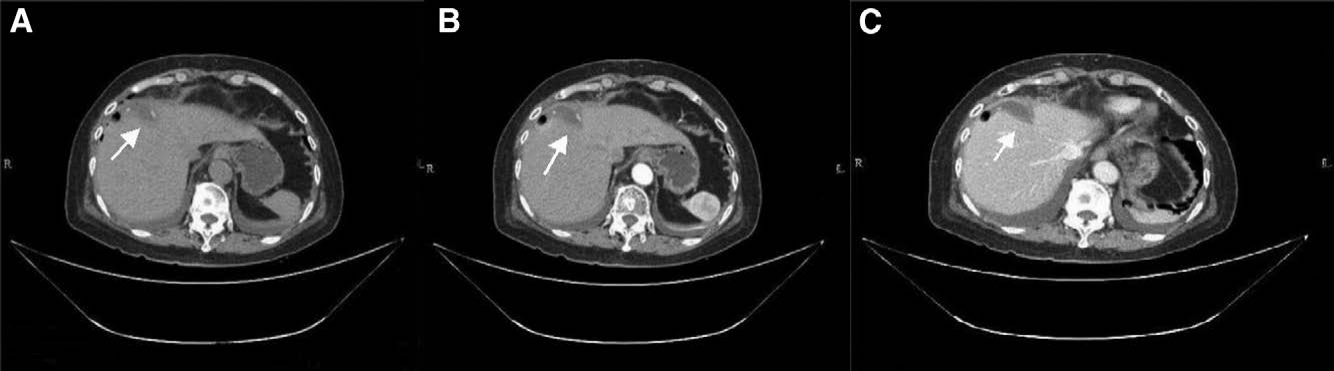
Preoperative CT images. (A) Plain CT scan showing low-density circular nodules in the left anterior lobe of the liver. (B) CT arterial phase enhancement showed mild enhancement. (C) CT portal phase enhancement showed a mild degree of enhancement, which was low density relative to normal liver tissue. CT = computed tomography.

**Figure 2.**
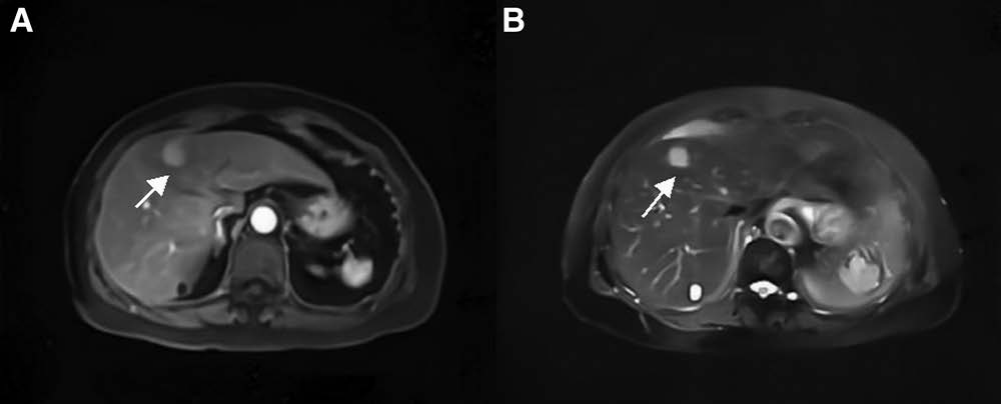
Preoperative MRI. (A) Dynamic enhancement of the mass showed low signal in T1-weighted images. (B) T2-weighted images and diffusion-weighted images showed high signal areas in the lesion. MRI = magnetic resonance image.

**Figure 3.**
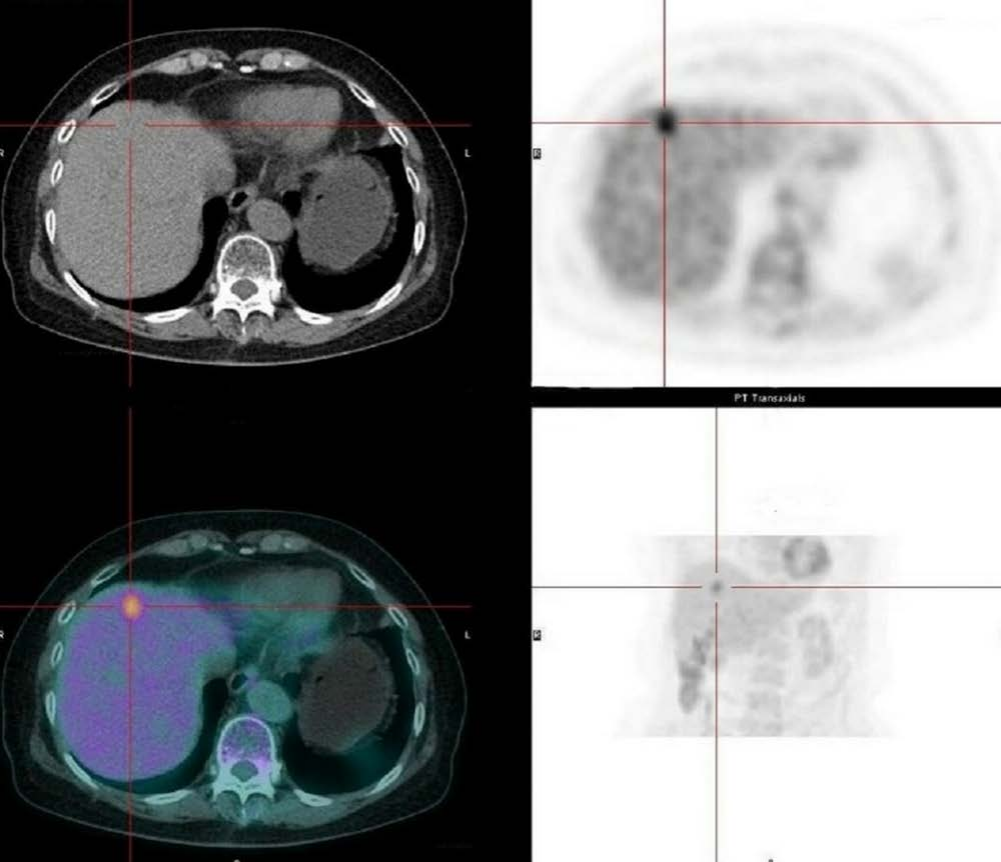
A slightly low-density lesion with increased FDG uptake was seen at the upper margin of the left medial lobe. FDG = fluorodeoxyglucose.

A 77-year-old female was admitted to the cardiology department of our hospital due to “repeated paroxysmal palpitations for 8 years, aggravated for more than 1 month.” She had no significant medical history or remarkable family history. Echocardiogram showed mitral valve and tricuspid valve mild regurgitation. No obvious thrombus was observed in left atrial by contrast-enhanced ultrasound. The initial diagnosis was paroxysmal AF, which improved after symptomatic treatment with drugs. Three days later, enhanced CT of the whole abdomen indicated a slightly hypodense focal mass in segment IVA of the liver, with a diameter of about 14 mm and a plain CT value of about 50 Hu, and multiple cysts in both kidneys (Fig. 1). Enhanced magnetic resonance imaging of the upper abdomen suggested the lesion appeared to have low intensity on T1-weighted imaging, slightly high intensity on T2-weighted imaging. And diffusion-weighted imaging showed decreased diffusion (Fig. 2). Considering them above, she was transferred to the hepatobiliary and pancreatic surgery department. Physical examination was unremarkable without arrhythmia, abdominal tenderness or other positive signs. The viral markers were HBsAg (−), HBsAb (−), and anti-HCV (−). Laboratory tests excluding glomerular filtration rate was within normal range. Tumor bio-markers such as alpha fetoprotein, carcinoma-embryonic antigen and carbohydrate antigen 19-9 were unremarkable. Total positron emission tomography-computed tomography imaging indicated slightly low-density lesions with increased glucose metabolism at the upper margin of the left medial lobe (Fig. 3). Additional diagnosis was as follows: left hepatic space occupying lesion and renal insufficiency.

**Figure 4.**
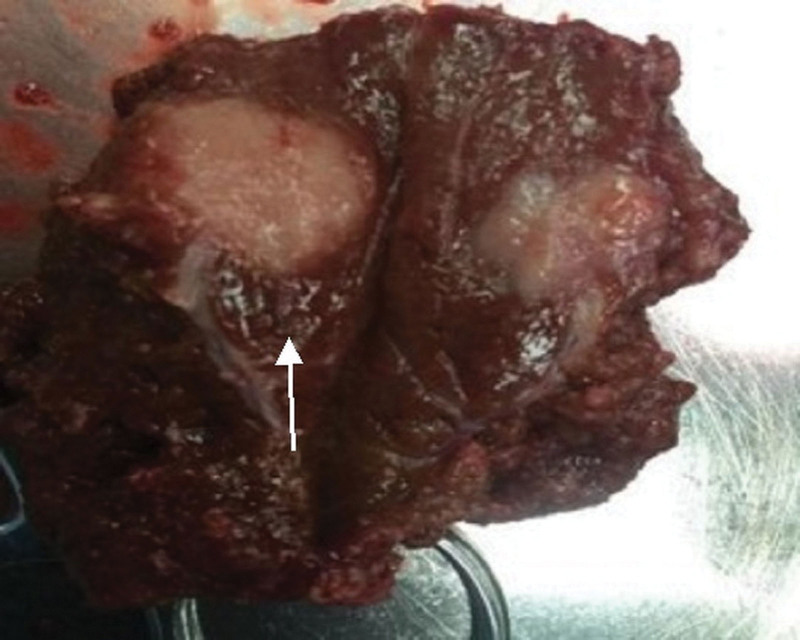
Surgical resection of specimen (scale bar in cm).

**Figure 5.**
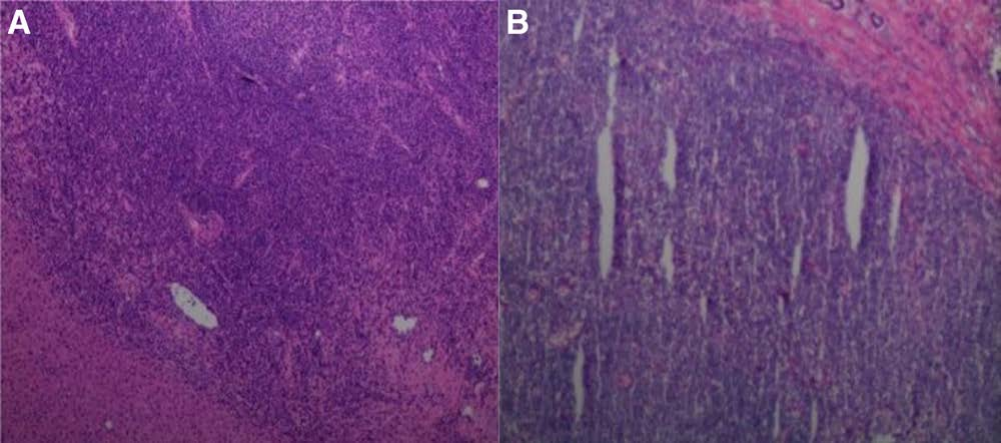
(A and B) Immunohistochemical analysis showed the normal tissue structure of local liver tissue disappeared and more lymphocyte nodular distribution could be seen (original magnification, ×200).

According to the imaging examination, the lesion was considered to be malignant. After full communication with the patient and his family, it was decided to perform surgical resection of the tumor to clarify its pathological nature owing to the extremely suspicious diagnosis. Intraoperative findings suggested the lesion was found in segment IVA of the liver, about 2.5 × 2 cm in size and irregular in shape. The cut surface was fish-like, about 2 × 1.5 cm in size (Fig. 4). Therefore, laparoscopic partial hepatectomy and abdominal lymphadenectomy were performed. Some liver masses were resected and sent for pathological examination, which suggested active hyperplasia of small B-lymphoid cells and lymphoid follicular hyperplasia and plasma cell differentiation. B-cell non-Hodgkin lymphoma in the outer margin area of lymphoid tissue was considered. Immunohistochemical findings were positive for CD20, Ki-67 and BCL-2, whereas negative for CD3, CD5, CD10, and TDT (Fig. 5). Based on the above pathological features, the patient was diagnosed as PHL, which was considered to be low-grade malignant B-cell lymphoma in the outer edge of the node. Postoperative recovery was good, no obvious abnormalities were found in the enhanced CT of the whole abdomen after the operation, and all examination indexes were gradually normal. She was discharged from hospital and regularly followed up.

After discharge, he continued to receive metoprolol sustained-release tablets to control ventricular rate and warfarin to anticoagulate. Regular hematology follow-up and review of relevant examination indicators (including coagulation function, β2 microglobulin, abdominal ultrasound, neck and supraclavicular ultrasound) were conducted.

## 3. Discussion

In recent years, PHL-related reports have been gradually attracting clinical attention. The etiology and pathogenesis of PHL are still unclear. According to relevant literature reports, the main causes of PHL include hepatitis B, hepatitis C, or Epstein-Barr virus infection, and immune dysfunction, such as AIDS and organ transplantation, especially the former.^[3]^ MALT lymphoma usually occurs in mucosa-associated exodal organs, mostly in gastrointestinal tract, eye, skin, thyroid, lung, mammary gland and etc, while primary hepatic MALT lymphoma is rare, and it is still rarely reported in recent years.^[4]^ Common clinical manifestations of primary hepatic MALT lymphoma include right upper abdominal discomfort, nausea or vomiting, general fatigue, lymphoma “B” symptoms (fever, night sweats, weight loss, etc) and enlarged liver can be detected in about 50% of patients on physical examination.^[5]^ In addition, a small number of patients may have jaundice, thrombocytopenia and other symptoms,^[6]^ so it is easily confused with hepatocellular carcinoma, intrahepatic bile duct carcinoma, hepatic hemangioma, hepatic metastasis and hepatic abscess in clinical practice.^[7]^ The first peculiarity of our case is that the patient developed none of these symptoms or signs above.

Imaging examination plays an important role in the early diagnosis of primary hepatic MALT lymphoma. Ultrasound examination has the advantages of convenience, noninvasive and economical benefits. It is often used as the first screening method, manifested as the lesion with low echo, unclear boundary and irregular shape.^[4]^ Most primary hepatic MALT lymphoma presents low-density lump on CT plain scan, and it is evenly and mildly enhanced after third-stage dynamic enhancement, which is consistent with the pathological characteristics of hepatic lymphoma. Some patients can see typical “vascular floating sign,” that is, blood vessel passing through the tumor, without signs of stenosis and invasion in the blood vessel itself.^[8]^ Based on magnetic resonance imaging plain scan, primary hepatic MALT lymphoma shows low or isosignal signal on T1WI, medium to high signal on T2WI and high signal on diffusion-weighted imaging,^[4]^ almost similar to our case. PET/CT can show the metabolic characteristics of tissues and organs. Malignant tumor cells have higher ^18^F-FDG intake than normal tissue cells.^[5,9]^ In this case, slightly low-density focus with increased glucose metabolism at the upper margin of the left medial lobe can be seen. However, definitive diagnosis still requires the histological results obtained by ultrasound or CT-guided liver biopsy or surgical resection.^[8]^

MALT lymphoma has great heterogeneity in treatment, and there is no universally accepted standard. Treatment selection mainly depends on 2 aspects: primary site and spread site. The main treatments reported in the literature include chemotherapy, radiation therapy, surgical resection of the primary lesion, immunotherapy, etiological therapy and target therapy. Currently, R-Benda regimen is the preferred chemotherapy regimen for non-Hodgkin lymphoma, which is used as the standard regimen for the treatment of various types of chronic lymphomas.^[10]^ However, in existing and previous studies, there is no data to support adjuvant chemotherapy and/or immunotherapy to benefit postoperative patients. Similarly, adjuvant radiotherapy is not required after complete resection. For specific patients, such as elderly patients, patients with severe diseases or complete resection of the lesion or asymptomatic even inert diseases, the “wait and watching” strategy is recommended,^[10]^ which is basically consistent with the treatment of our case – surgical resection of the lesion, acquisition of pathological diagnosis, no additional postoperative intervention and regular follow-up.

The presence of this patient complicated with AF is the second particularity of our case, but there are few studies related to it at present, and only one MALT lymphoma complicated with AF has been reported, the primary site of which is the lung. Marc Sorigue et al^[11]^ found that the incidence of AF in patients with non-Hodgkin lymphoma was higher than that of the general population, which may be attributed to the complications of the disease itself and/or the use of chemotherapy drugs. For example, R-CHOP, a chemotherapy protocol commonly used by MALT lymphoma, contains anthracyclines. The fatal side effect is cardiotoxicity. A prospective study concluded that increased cumulative anthracycline dose and the occurrence of AF were independently associated with all-cause mortality. In detail, patients with AF in non-Hodgkin lymphoma had a higher incidence of acute heart failure than those without AF (40% vs 3.8%; *P* < .001), new AF may predict poor prognosis after anthracycline chemotherapy in patients with malignant lymphoma.^[12]^ It is well known that anticoagulation is one of the cornerstones of the treatment of AF. Our patient was routinely anticoagulated with warfarin after surgery and was regularly tested for international normalized ratio and prothrombin time. However, a recent review suggested that the most fundamental issues in anticoagulation included the assessment of adverse interactions between anticoagulants and anticancer drugs because of the risk of bleeding.^[13]^ There is insufficient evidence to prove the efficacy and safety of novel direct oral anticoagulants (DOACs) and vitamin K antagonists in cancer patients with AF.^[14]^ But a recent meta-analysis on the efficacy and safety of DOACs and vka in oncology found that DOACs were more effective in preventing stroke during AF,^[15]^ which provided a reference for the subsequent treatment of patients.

After 20 months of follow-up, the patient’s disease was stable. Although the postoperative ultrasonography of the patient suggested supraclavicular lymph node enlargement, the boundary was clear and no lymphatic or vascular invasion was found. The patient belongs to the elderly group, accompanied by paroxysmal AF, hyperlipidemia, renal insufficiency and other underlying diseases. Therefore, it is temporarily not given due to the possibility of serious adverse reactions due to renal toxicity and cardiotoxicity of chemotherapy.

However, our study has 2 limitations. First, the patient was followed up for a short period of only 20 months. The patient’s subsequent treatment plan may have to be adjusted as appropriate. Second, our study was an exploratory one. Because of the presence of AF, we did not proceed with chemotherapy and opted for a “watch-and-wait” strategy. At present, the treatment plan for MALT patients combined with AF has not been fully confirmed. Future randomized controlled trials in this population are expected.

## 4. Conclusions

In summary, the incidence of lymphoma is low, the clinical symptoms and the imaging examinations are nonspecific. For patients with unclear diagnosis, clinical thinking should not be limited and primary hepatic MALT lymphoma should be included in the identification category. Needle biopsy or surgical resection is necessary for definitive diagnosis. Moreover, for patients with primary hepatic MALT lymphoma combined with AF, toxicity caused by combined chemotherapy should be fully considered, careful selection should be made based on the general conditions and complications of patients so as to formulate a good treatment plan for patients to accelerate oncological recovery.

## Acknowledgments

We would like to thank all of our colleagues in the medical and nursing team of the hepatobiliary and pancreatic surgery.

## Author contributions

**Data curation:** Chenming Liu, Jiayao Zhang, Yingjie Chai.

**Formal analysis:** Chenming Liu, Jiayao Zhang.

**Supervision:** Haijun Tang.

**Writing – original draft:** Chenming Liu, Yuxing Liu.

**Writing – review & editing:** Baochun Lu, Haijun Tang.
